# A thin layer of sucrose octasulfate protects the oesophageal mucosal epithelium in reflux oesophagitis

**DOI:** 10.1038/s41598-019-39087-4

**Published:** 2019-03-05

**Authors:** Takuya Hayakawa, Shizuka Kawasaki, Yutaka Hirayama, Takuya Tsutsui, Eiji Sugiyama, Kiyo Adachi, Ryo Kon, Makoto Suematsu, Yuki Sugiura

**Affiliations:** 1 Research & Development Headquarters, Pharmaceutical Research Laboratories, Lion Corporation, 100 Tajima, Odawara, Kanagawa 256-0811 Japan; 2 Research & Development Headquarters, Analytical Technology Research Center, Lion Corporation, 7-2-1 Hirai, Edogawa-ku, Tokyo, 132-0035 Japan; 3 Department of Biochemistry, Keio University School of Medicine, 35 Shinanomachi, Shinjuku-ku, Tokyo 160-8582 Japan

## Abstract

Sucralfate is effective for the treatment of gastric and duodenal ulcers owing to its protective gel-forming ability. However, the mechanism by which sucralfate protects the oesophageal mucosa against reflux oesophagitis has not been clarified. We aimed to investigate the mechanisms of action of sucralfate and sucrose octasulfate (SOS), a component of sucralfate. SOS and sucralfate were administered to oesophagitis-induced rats, and the ulcer lesion size was macroscopically examined and scored. Effective pepsin activity in the gastric juices obtained from the animal model was evaluated by a casein digestion test. Sucralfate and SOS improved the pathology scores in a dose-dependent manner, whereas gastric juice pepsin activity was not impaired by therapeutic doses of SOS. As SOS lacks the ability to form a thick gel layer by polymerisation, we examined the distribution of SOS in the mucosal lumen by imaging mass spectrometry (IMS) to determine whether SOS directly adheres to the mucosal surface. A clear homogeneous thin-layer SOS film (>100 μm thick) was visualized on the oesophageal mucosal surface. Moreover, this SOS film formation was enhanced at ulcer lesion sites. Taken together, SOS appears to protect oesophageal mucosa against reflux oesophagitis via thin-layer formation on the mucosal surface.

## Introduction

Sucralfate, a complex salt composed of aluminium hydroxide and sucrose octasulfate (SOS) polymer, is widely used to treat gastrointestinal diseases such as gastric and duodenal ulcers. Sucralfate is effective for the treatment of human reflux oesophagitis^1–4^; however, current first-line treatments for gastroesophageal reflux disease (GERD) are proton pump inhibitors (PPIs) which control gastric acid secretion. More recently, sucralfate or a combination of sucralfate and acid suppressor has offered new therapeutic regimens for patients with unsatisfactory responses to PPIs alone^5^; these are especially preferred by patients during pregnancy and lactation^6,7^.

Under acidic conditions, sucralfate polymerizes to a viscous adhesive gel, which adheres to inflammatory sites and creates a strong protective gel layer against pepsin, acid, and bile acid. Gel formation is considered to be the main mechanism for the effects of sucralfate on gastric and duodenal ulcers^8–12^. However, the duration of drug passage through the oesophagus is much shorter than that of the stomach and duodenum. Moreover, unlike in digestive organs, lumen pH in the oesophagus is nearly neutral, rendering it difficult for the drug to form a gel. Hence, the mechanisms underlying the efficacy of sucralfate against reflux oesophagitis requires clarification.

In a series of studies, Orlando and colleagues attempted to determine the protective mechanisms of sucralfate against reflux oesophagitis^13–16^, focusing on the main chemical component of sucralfate, SOS, which lacks the ability to form a gel. Their research showed that SOS administration is effective in models of oesophagitis because SOS is able to suppress H^+^ ion permeability in biopsied human oesophageal mucosa. However, it is still unclear whether SOS directly adheres to the mucous surface.

Imaging mass spectrometry (IMS), an emerging technique, does not require chemical probe or labelling and allows the analysis of spatial distribution of small molecules, including administered drugs^17^. IMS has therefore been applied in pharmacokinetic studies^18^. We aimed to investigate the distinct mechanisms of sucralfate and SOS in an oesophagitis model. Although we initially hypothesized that mucosal protection by sucralfate and SOS was mediated via the inhibition of digestive enzymes, gastric juice pepsin activity was not impaired by therapeutic doses of SOS. We therefore utilized IMS to visualize the localisation of SOS within oesophageal tissues to test the hypothesis that SOS directly adheres to the mucosal surface. The obtained images clearly demonstrate that a thin layer of SOS was tightly attached to the surface of the oesophageal mucosa, especially within ulcer sites, in an oesophagitis model.

## Materials and Methods

### Drugs and reagents

Sucrose octasulfate sodium salt (Toronto Research Chemicals, Inc., Toronto, ON, Canada) was dissolved in distilled water for all experiments and was administered orally at a volume of 2.5 mL/kg of body weight. The following drugs and reagents were also utilized: isoflurane inhalation solution (Pfizer, New York, NY, USA); 10% sucralfate-containing suspension (ULCERLMIN® Oral Suspension 10%; Chugai Pharmaceutical Co. Ltd., Tokyo, Japan); pepsin (1:10,000, from porcine stomach mucosa), casein, trichloroacetic acid, sodium carbonate, and Folin’s reagent (all purchased from Wako Pure Chemical Industries, Ltd., Osaka, Japan); and 9-aminoacridine (9-AA; Thermo Fisher Scientific K.K., Yokohama, Japan).

### Animals

All experiments utilized male Sprague–Dawley rats, weighing 250–300 g (SLC, Inc., Shizuoka, Japan). The animals were housed in a room maintained at 23 °C ± 1 °C under a 12-h light/dark cycle, with *ad libitum* access to water and food. On the day before the experiment, the animals fasted for 18 h and were kept in raised mesh-bottom cages to prevent coprophagy. All animal experiments were approved by the Animal Care and Use Committee of Lion Research Laboratories (Tokyo, Japan) and were conducted in accordance with the internal guidelines for animal experiments and the ethical policies of Lion Corporation.

### Esophagitis induction and drug administration

**Figure 1:**
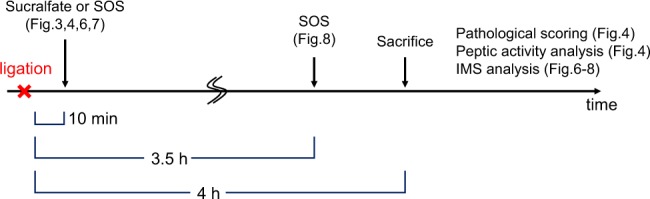
Time course of model rat generation and sucralfate or SOS treatment. First, a rat model of reflux oesophagitis was prepared by forestomach and pylorus ligation. Sucralfate or SOS was then administered to evaluate the therapeutic effect of these drugs (Fig. 3). Using the same samples used to evaluate gross pathology, an IMS analysis was performed to evaluate whether SOS adhered to the mucosal surface (Figs 6 and 7). Finally, we evaluated the localization of SOS administered after severe inflammatory lesions were induced (Fig. 8). SOS, sucrose octasulfate; IMS, imaging mass spectrometry.

An overview of the experiment is provided in Fig. 1. A model of rat reflux oesophagitis was prepared by forestomach and pylorus ligation, as described in previous reports^19,20^. Under isoflurane anaesthesia, pylorus and the limiting ridge (transitional region between the forestomach and corpus) were ligated. Sucralfate or SOS (in doses of 1.8–144 μmol/kg) was then administered via the oesophagus for transport to the stomach at 10 min or 3.5 h after ligation to evaluate early- and late-phase therapeutic drug effects, respectively. Four hours later, the gastric juice refluxed into the oesophagus, causing severe erosion. The oesophagus was then removed, dissected along the long axis, and spread onto filter paper. For IMS assessments, oesophageal samples were immediately frozen with powdered dry ice and stored at −80 °C until analysis. Control oesophageal tissues were prepared from normal rats that did not undergo the ligation operation.

### Gross Pathology

The total areas (mm^2^) of the oesophageal lesions were measured under a stereoscopic microscope and the gross pathology scored as follows: 0, no visible lesions; 1, mucosal erosion; 2, total area of mucosal damage, including an ulcer, was <30 mm^2^; 3, total area of mucosal lesions, including an ulcer, was ≥31 mm^2^; and 4, perforation. The ability of sucralfate and SOS to improve the pathology of oesophagitis was evaluated.

### Measurement of peptic activity *in vivo*

We initially hypothesized that mucosal protection by sucralfate and SOS was mediated via the inhibition of digestive enzymes. To test this hypothesis, the effective peptic activity in the gastric juice from the rat model was assessed by a digestion test using casein as a substrate. Peptic activity was assessed *in vivo* using the supernatants of rat gastric juices collected 4 h after ligation; we utilized a modified method described in the Japanese Pharmacopeia, 16th Edition, with casein as a substrate^21^. Briefly, the collected gastric juices were diluted 50-fold with 0.04 mol/L hydrochloric acid. A 0.1 mL volume of the diluted juice was then added to 0.5 mL of substrate solution (0.72% lactic acid, 0.6% casein; pH 2.0), and the mixture incubated at 37 °C for 10 min. The casein digestion reaction was stopped with 5% trichloroacetic acid, followed by neutralisation with a 0.55 mol/L sodium carbonate solution. Tyrosine and tyrosine residues from casein were detected by the addition of Folin’s reagent.

### Measurement of peptic activity *in vitro*

The effect of SOS on peptic activity was further tested using artificial gastric juice. In this test, we evaluated whether SOS acts on the substrate or pepsin by pre-incubating the casein substrate or pepsin with SOS in the artificial gastric juice. The 50% inhibitory concentration (IC_50_) value for pepsin was estimated.

SOS was pre-incubated with the substrate as described below. An equal volume of 10 to 240 μmol/L SOS was added to 500 μL of a substrate solution (1.44% lactic acid, 1.2% casein; pH 2.0), and the mixture incubated at 37 °C for 30 min. Subsequently, the artificial gastric juice [0.24% hydrochloric acid, 0.2% sodium chloride, 0.85% pepsin (1:10,000); pH 1.2] was diluted 50-fold with 0.04 mol/L hydrochloric acid and 0.1 mL of the diluted juice added to the reaction solution. The mixture was added to 0.5 mL of the supernatant and reacted at 37 °C for 10 min. The casein digestion reaction was stopped with 5% trichloroacetic acid, followed by neutralisation with a 0.55 mol/L sodium carbonate solution. The degradation products were quantitated by a colorimetric reaction using Folin’s reagent.

Similarly, SOS was pre-incubated with pepsin in the artificial gastric juice. An equal volume of 10 to 240 μmol/L SOS was added to 100 μL of the artificial gastric juice [0.48% hydrochloric acid, 0.4% sodium chloride, 1.7% pepsin (1:10,000); pH 1.2], and the mixture incubated at 37 °C for 30 min. Subsequently, the supernatants for the artificial gastric juice, reacted with SOS, were diluted 50-fold with 0.04 mol/L hydrochloric acid. A 0.1 mL volume of the diluted supernatant was then added to 0.5 mL of a substrate solution (0.72% lactic acid, 0.6% casein; pH 2.0) and reacted at 37 °C for 10 min. The casein digestion reaction was measured in the same manner described for SOS pre-incubated with substrate.

### Imaging mass spectrometry

While sucralfate can form a thick gel layer inside the gastrointestinal tract, SOS lacks this gel-forming ability^22^. Thus, by using IMS, we tested the hypothesis that SOS might form an alternative protective structure by directly adhering to the mucosal surface. Additionally, we evaluated the ability of SOS to accumulate at inflammation sites.

Thin sections (12-μm thick) of the oesophageal samples were prepared at −21 °C using a cryostat (CM 1850; Leica, Wetzlar, Germany), and thaw-mounted on conductive indium–tin oxide glass slides (20 Ω, SI0020N; Matsunami Glass Industry Co. Ltd.). A matrix was prepared using a solution of 10 mg of 9-AA dissolved in 1 mL of 80% ethanol^23^. The 9-AA matrix solution was manually sprayed onto the samples using an airbrush (Procon Boy PS 270 WA Platinum; Mr. Hobby), until the tissue surface became uniformly light yellow in colour.

**Figure 2:**
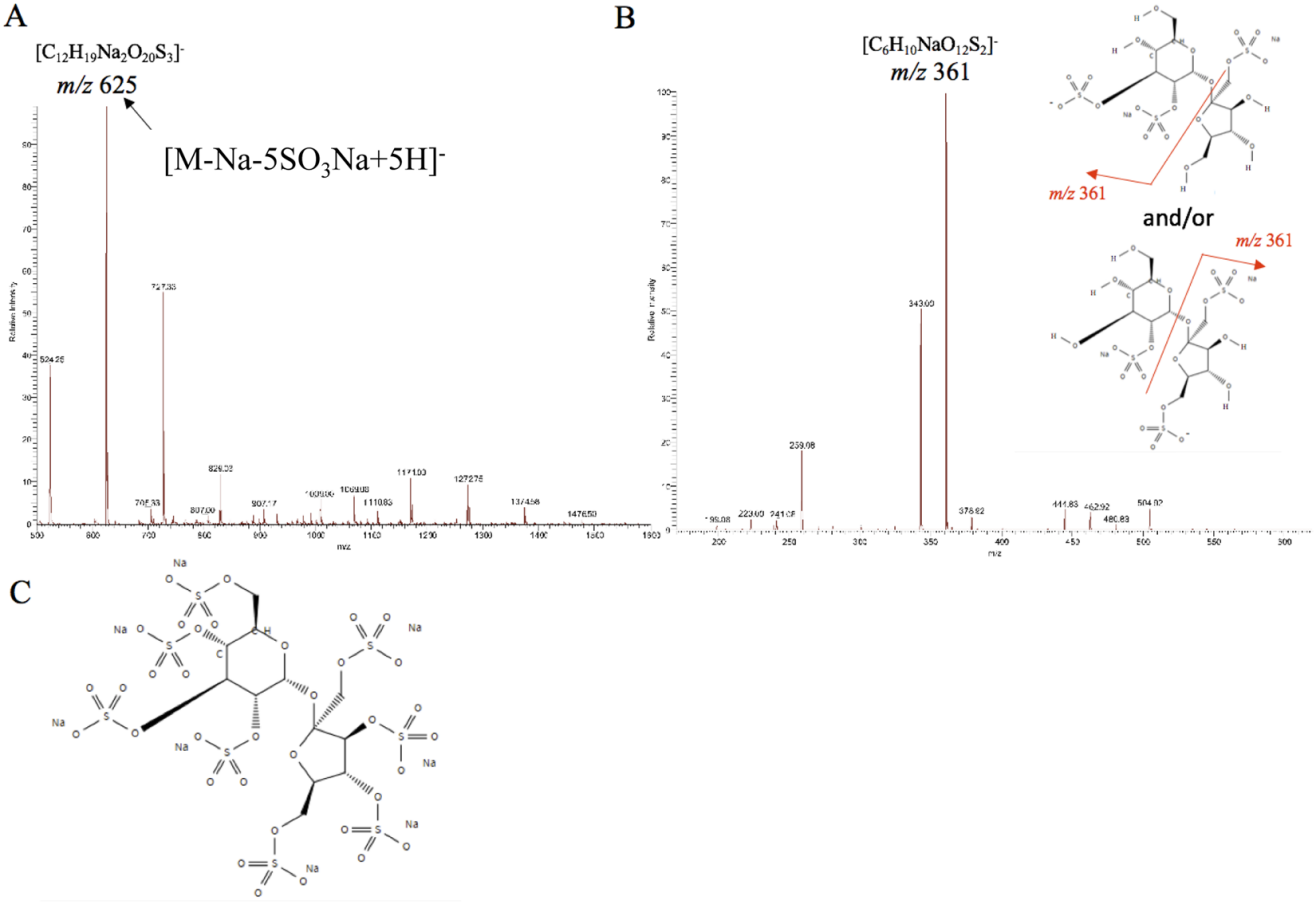
Development of a specific SOS detection method by tandem MS. Qualitative analysis of an SOS-Na reference compound spotted on oesophageal tissues was performed using a MALDI LTQ-XL instrument. (**A**) On the MS spectrum (*m/z* 500–1600), the most intensive ion at *m/z* 625 was identified as ([M-Na  − 5SO_3_Na + 5 H]^-^); (**B**) Product ion spectrum (*m/z* 170–620) for the ion at *m/z* 625; (**C**) Structure of SOS-Na (molecular weight: 1157.628). MALDI, matrix-assisted laser desorption/ionization; MS, mass spectrometry; SOS, sucrose octasulfate.

Mass spectrometry (MS)/MS-based IMS was performed using a matrix-assisted laser desorption/ionisation (MALDI) LTQ-XL instrument (Thermo Fisher Scientific)^24^. To conduct the IMS experiment with adequate signal selectivity that could exclusively detect the SOS signal from complex tissue samples, we had to determine SOS-specific ion signals on MALDI–MS/MS. For this purpose, we measured an authentic SOS standard spotted on an oesophageal section (see Fig. 2). In the mass spectrum, a molecular ion at *m/z* 625 ([M-Na − 5SO_3_Na + 5 H]^-^) was detected, having the highest intensity (Fig. 2A). We then performed MS/MS targeting the ion at *m/z* 625. In the obtained product ion spectrum, a strong product ion at *m/z* 361 was detected with the highest sensitivity (Fig. 2B). Thus, during subsequent imaging assessments, an ion with *m/z* 625 was used as the parent ion, with an isolation window of *m/z* 1.0; the mass range from *m/z* 360 to 363 was recorded for each data point. The collision energy was 25% of the maximum available energy for the LTQ-XL, and the laser energy was set to 30 µJ. The scanning pitch of the laser irradiated area was 75 μm. Image reconstruction was performed using Image Quest software (Thermo Fisher Scientific, Waltham, MA).

### Statistical analysis

Data are expressed as mean ± standard error of the mean (SE). Differences in gross pathology were evaluated using Steel’s multiple comparison test, and analysed using JMP Pro11 software (SAS, USA). IMS data was evaluated using a region of interest (ROI) analysis; regional intensities of the SOS-selective signal (*m/z* 625 > 361) were calculated in mucosal and muscle regions using ImageJ software^25^. Group differences were assessed using the Student’s *t*-test. For all analysis, *p* values < 0.05 were considered statistically significant.

## Results

### Sucralfate and SOS improve the gross pathology of reflux oesophagitis

**Figure 3:**
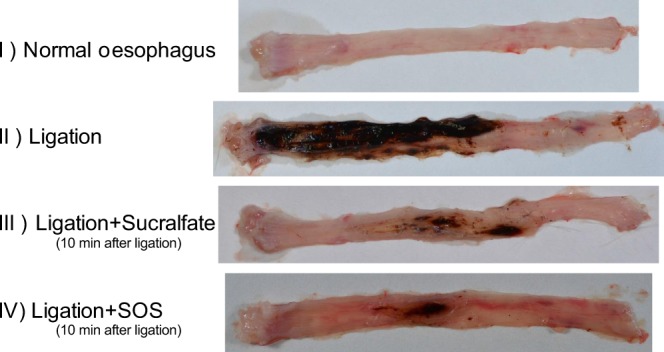
Sucralfate and SOS administration attenuate oesophagitis pathology. Images of the gross pathology of the oesophagus in the rat reflux oesophagitis model (forestomach and pylorus ligation) for the following experimental groups: Normal oesophagus (without ligation), I; Ligation (without drug administration), II; Ligation + preventive administration of Sucralfate (3.6 μmol/kg), III; Ligation + preventive administration of SOS (3.6 μmol/kg), IV. SOS, sucrose octasulfate.

Macroscopic observations of oesophageal tissues obtained from reflux oesophagitis modelled-rats clearly demonstrated that both compounds improved the gross pathology of reflux oesophagitis (Fig. 3). While the control tissue exhibited severe oesophagitis, namely bleeding, mucosal erosion, and perforation (Fig. 3-I), the development of pathological lesions was apparently attenuated by early sucralfate and SOS administration (Fig. 3-III, IV, respectively).

**Figure 4:**
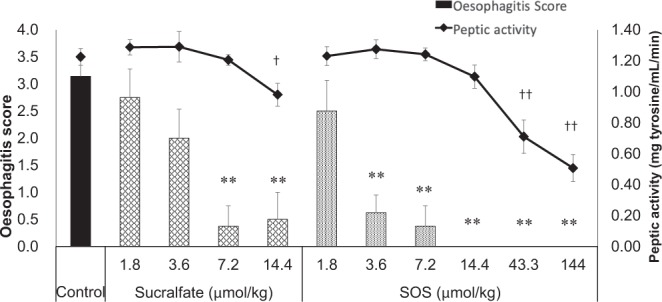
SOS exhibited stronger therapeutic effect than sucralfate at low doses against reflux oesophagitis. The oesophageal pathology scores (left ordinate) and peptic activity in gastric juices collected 4 h after the ligation (right ordinate) are shown for control and sucralfate- and SOS-administered reflux oesophagitis model rats. The drugs were administered 10 min after ligation. Data are expressed as mean ± SE for eight animals. **Significantly different from the oesophagitis score of the control animals at p < 0.01. ^††^Significantly different from the peptic activity of the control animals at p < 0.05 and p < 0.01 (sucralfate and SOS, respectively). These data were evaluated using Steel’s multiple comparison test. SOS, sucrose octasulfate; SE, standard error.

Moreover, we found that the two compounds attenuated oesophagitis pathology in a dose-dependent manner (Fig. 4, bar graphs). As expected, the control group had the highest pathological score (mean, 3.14); the administration of sucralfate however reduced the score in a dose-dependent manner (score values: 2.75, 2.00, 0.38, and 0.50 for the 1.8, 3.6, 7.2, and 14.4 μmol/kg dosage groups, respectively). SOS administration also resulted in a dose-dependent attenuation of the pathological score (scores: 2.50, 0.63, 0.38, and 0.00 for 1.8, 3.6, 7.2, and 14.4 μmol/kg dosage groups, respectively). Interestingly, the therapeutic performance of SOS was even stronger than that of the sucralfate, especially among low doses; only SOS led to a significantly reduced pathological score compared to that in the controls at a dose of 3.6 μmol/kg.

### Low doses of sucralfate and SOS did not affect peptic activity in gastric juices

**Figure 5:**
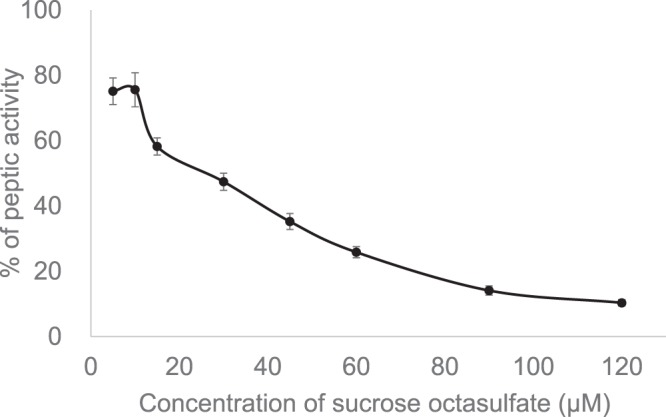
Low doses of SOS had no effect on peptic activity in gastric juices. The plot depicts the inhibition of peptic activity by SOS pre-incubated with casein substrate. Data are expressed as mean ± SE of three replicates. SOS, sucrose octasulfate; SE, standard error.

Line plots in Fig. 4 show the results of the digestion test using casein as a substrate. As shown, SOS did not significantly reduce peptic activity at the lower therapeutic doses, 3.6–14.4 μmol/kg. The *in vitro* assessments of peptic activity demonstrated that SOS inhibited peptic activity at concentrations of 5–120 μmol/L; the estimated IC_50_ value was 25.4 μmol/L (Fig. 5). These results depicting that the inhibition of digestive enzymes is not a mechanism of the protective effect of SOS led us to evaluate SOS drug delivery to the oesophageal mucosa.

### Administered SOS forms a thin layer in the oesophageal mucosal lumen

**Figure 6:**
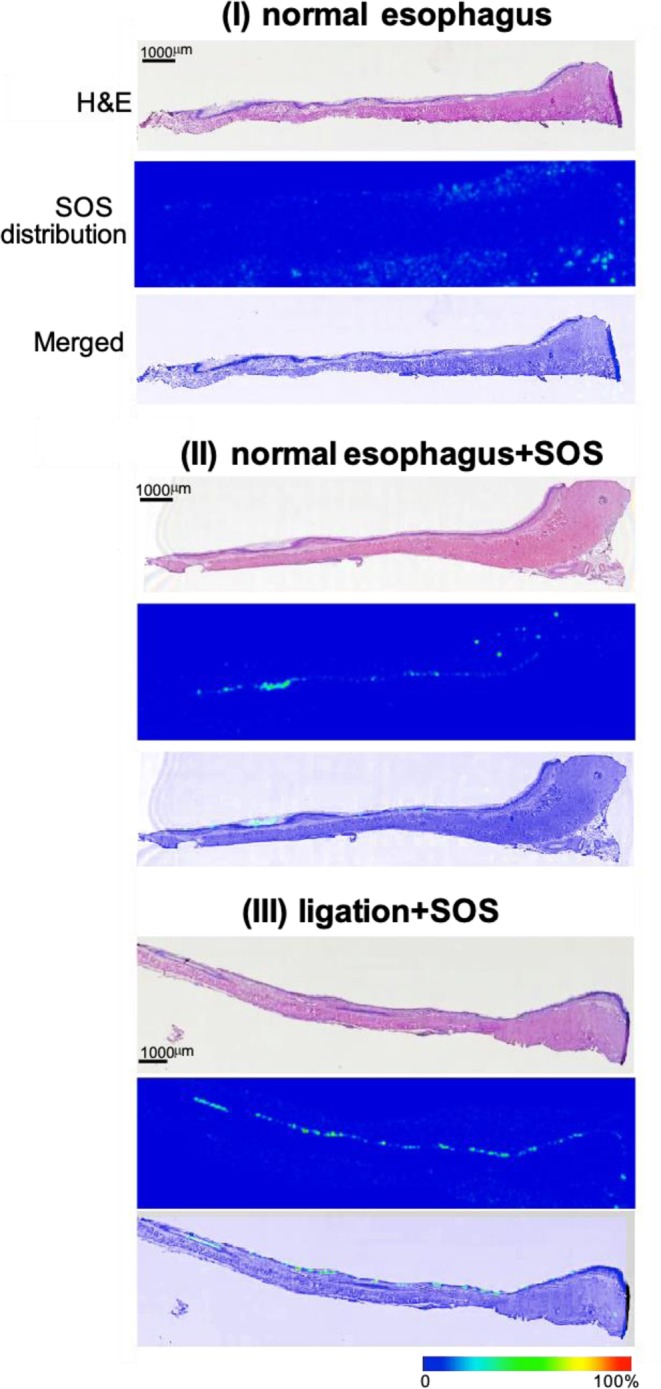
Administered SOS formed a thin layer in the oesophageal mucosal lumen. Representative images of the localization of SOS on the oesophageal mucosal surface using IMS. Imaging was performed for (I) normal group (without ligation, group I in Fig. 1), (II) normal oesophagus + SOS 7.2 μmol/kg and (III) Ligation + preventive administration of SOS 7.2 μmol/kg (group IV in Fig. 1). Widespread signals are clearly detected in the oesophageal lumen. Bar = 1,000 µm. SOS, sucrose octasulfate; IMS, imaging mass spectrometry.

Having established the SOS-specific detection method by tandem MS (see methods; Fig. 2), we applied the method to the imaging analysis to visualize the localisation of SOS. We analysed control oesophageal tissues without SOS administration (*n* = 2) and oesophageal tissues of oesophagitis modelled-rats that were administered 7.2 μmol/kg SOS, 10 minutes after ligation (*n* = 4). Such early SOS treatment resulted in almost complete suppression of ulcer development (observed in haematoxylin and eosin (H&E) images stained after IMS measurement, Fig. 6, upper panel of I–III).

Interestingly, we found that the administered SOS directly adhered to the mucosal surface. While no signals were detected in the normal oesophagus without surgery or drug administration (Fig. 6-I), by merging the SOS distribution images with H&E-stained images, weak SOS attachment on the normal oesophagus was observed (Fig. 6-II). Moreover, a clear thin SOS layer (>100 μm) was observed in the oesophagus that received ligation surgery (Fig. 6-III) to coincide with the mucosal layer of the oesophagus. This SOS layer was distributed along with the whole oesophageal lumen in the longitudinal direction. Since the border edge of the SOS layer, located at the boundary of the mucosa and submucosa was clearly visible (Fig. 6), SOS was suggested to specifically adhere to the mucosal surface, forming a thin layer.

**Figure 7:**
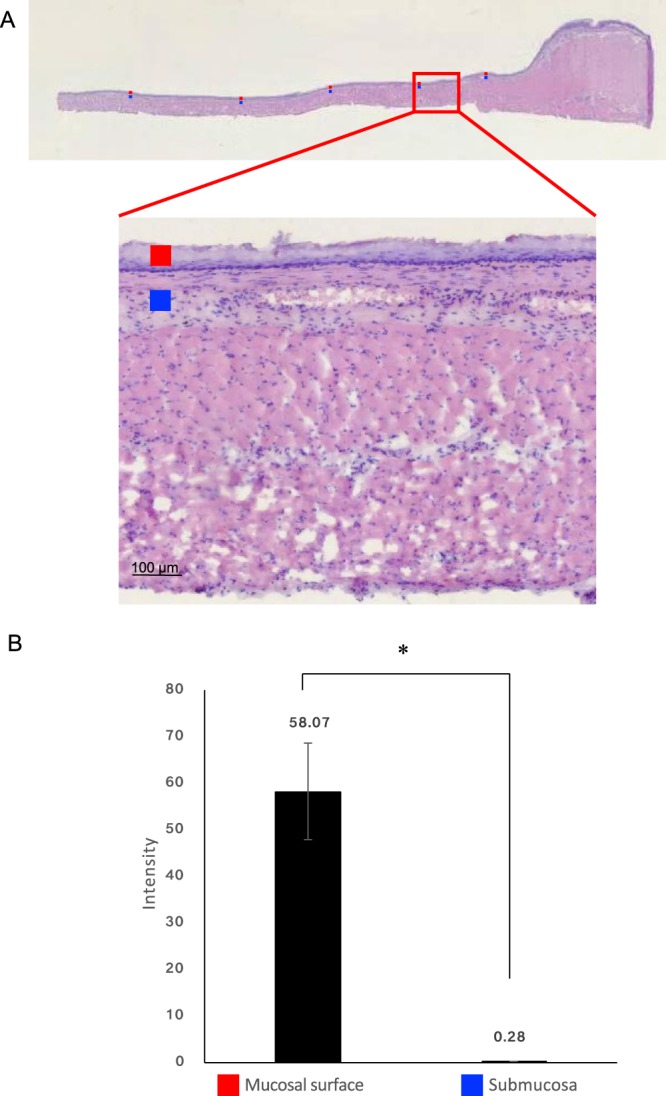
ROI analysis shows strong SOS accumulation on the mucosal surface. (**A**) Representative H&E-stained image of a thin section of the oesophagus. An ROI analysis of SOS ion intensity in the mucosal and muscle layers supports mucosal surface localisation. The spots represent ROI sites of mucosal surface and submucosal regions. (**B**) Bar graph depicts the comparison of SOS accumulation levels on the mucosal surface and muscle regions. *p < 0.01. Group differences were assessed using the Student’s t-test. SOS, sucrose octasulfate; ROI, region of interest, H&E, haematoxylin and eosin.

These observations were confirmed quantitatively (Fig. 7) in an ROI analysis where the SOS-derived signal intensity was compared between mucosal (red-coloured ROI) and submucosal layers (blue-coloured ROI). The SOS signal on the mucosal surface was more than 200-fold higher than that in the muscular layer.

### SOS strongly accumulates in inflammatory lesions

**Figure 8:**
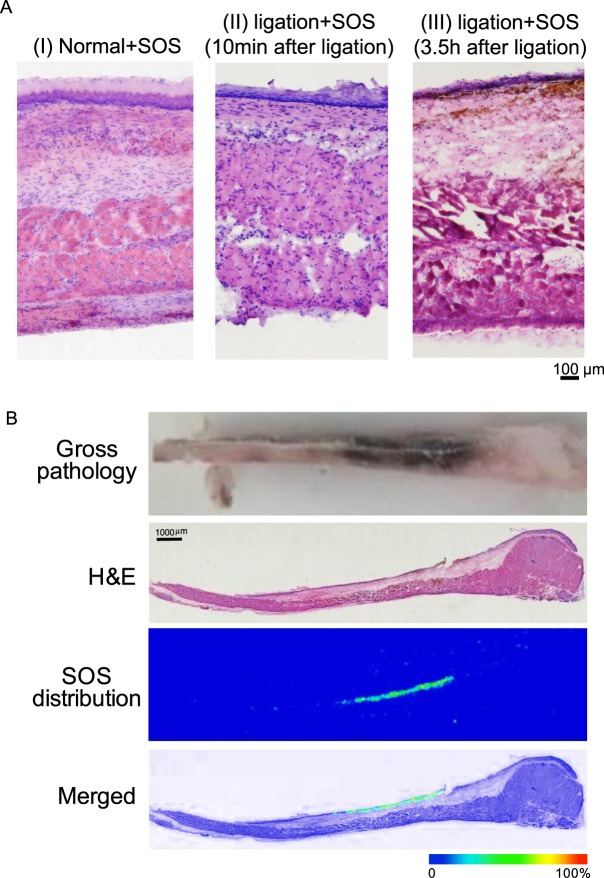
(**A**) SOS strongly accumulated in inflammatory lesions. H&E stained oesophagus sections from normal rat without treatment (I), oesophagus ligated rats followed by SOS administered at 10 min (II) and 3.5 h (III) after ligation. While early SOS treatment resulted in mild erosion and slight loss of mucosa, late administration could not attenuate these occurrences and show. (**B**) Representative images of the localisation of SOS in inflammatory oesophageal mucosal lesions using IMS. Imaging was performed with samples from the oesophagitis-induced rats administered 7.2 μmol/kg of SOS (n = 4). In the gross pathology image, inflammatory lesions appeared discoloured and black. Bar = 1000 µm. SOS, sucrose octasulfate; H&E, haematoxylin and eosin.

Thus far, we have shown that early SOS treatment resulted in thin-layer formation by adhering to the mucosal surfaces of oesophageal tissues. SOS may attenuate oesophagitis pathology, even when administered 10 minutes after ligation, as its preventive effect (Figs 6 and 7). High magnification H&E stained images proved the slight loss of mucosa which occurred homogeneously over the surface of the oesophagus (Fig. 8A-II). The thin-layered SOS may therefore interact with exposed cationic mucosal proteins.

Such observation led us to question whether SOS administered after large ulcer development (i.e., administration at several hours after ligation) could selectively accumulate at severe lesion sites. We examined the localisation of SOS administered after the induction of severe inflammatory lesions to evaluate the ability of SOS to accumulate at inflammatory sites. SOS (7.2 μmol/kg) was orally administered to the oesophagitis-induced rats (3.5 h after ligation, n = 4), and extracted tissues were analysed by IMS. SOS administration at this late time point, although attenuating oesophagitis pathology when compared to untreated oesophagus (no SOS; Fig. 3), loss of mucosal epithelium, haemorrhage and severe erosion were observed at the ulcer sites (Fig. 8A-III and gross pathology of Fig. 8B). As expected, the obtained IMS results showed that SOS strongly accumulated at the inflammatory lesion sites (Fig. 8B), demonstrating its ability to selectively produce a protective layer at inflammation sites.

## Discussion

In the present study, we aimed to evaluate the mucosal-protective ability and mechanisms employed by SOS and sucralfate in oesophagitis, using a rat reflux oesophagitis model. Since SOS lacks the ability to form a thick gel layer, which is the known main mucosal-protective mechanism of sucralfate, we focused on elucidating how SOS exhibits its therapeutic effect.

SOS is a component of sucralfate, which is an SOS–aluminium complex. In an ethanol-induced gastric injury model, the efficacy of SOS was reported to be weaker than that of sucralfate^26^. Additionally, an *in vitro* study showed that treatment with SOS alone cannot alleviate acid damaged-cultured cells^27^. However, several reports have indicated that SOS shows therapeutic effects in various oesophagitis models. For example, SOS suppressed H^+^ ion permeability in an electrophysiological experiment using biopsied human oesophageal mucosa^13^. In another report, SOS was shown to suppress mucosal injury in a rabbit oesophagitis model, which was induced by the reflux of artificial gastric juice^22^. Owing to these contradictory reports, the precise mechanism of SOS protective effects in reflux oesophagitis models still requires clarification.

In the present study, by using a rat reflux oesophagitis model, we demonstrated that SOS attenuates oesophagitis in a dose-dependent manner; SOS also exhibits an even stronger effect than sucralfate at several low dosage levels. The gross pathology, particularly the ulcer size, was significantly reduced, not only by sucralfate, but also by SOS administration.

Thus, in reflux oesophagitis, unlike in stomach injuries, a mechanism other than physical formation of a protective gel layer by sucralfate may exist. At first, we hypothesized that SOS impairs digestive enzyme activity, contributing to its therapeutic ability in oesophagitis. To test this hypothesis, we measured peptic activity in the gastric juice collected from control and model rats. Peptic activity was impaired only when the concentration of SOS was much higher than the therapeutic dose range. A large gap was observed between the therapeutic SOS dose range (3.6–14.4 μmol/kg) and the peptic activity reduction range (43.3–144 μmol/kg).

To obtain more specific details, we examined whether the anti-peptic activity of SOS is a substrate-protective effect or a reflection of enzymatic activity suppression. Based on *in vitro* pepsin activity measurements, SOS did not directly affect pepsin in the artificial gastric juice, whereas peptic activity was reduced in a dose-dependent manner when the substrate was pre-incubated with SOS. These results are consistent with the findings reported by Schweitzer *et al*.^22^. and Nagashima *et al*.^28^.

We then examined whether SOS could directly affect the mucosal surface. SOS has been reported to aid in the maintenance of mucosal tissue integrity and reduce hyperpermeability to mannitol in an oesophageal biopsy sample exposed to HCl^14^. This implies that SOS directly affects the mucosal surface, likely by forming a protective structure. However, there are no reports showing the distribution of SOS in oesophageal tissues as a molecular imaging technique capable of visualizing such small molecules was previously unavailable. With the development of IMS, we were able to examine whether SOS adhered to the mucosal surface or was incorporated into the inner wall of the oesophagus.

We found that SOS administered at an early time point (i.e., 10 minutes after ligation) formed a homogeneous thin layer (>100 μm) on the oesophageal mucosal surface. Since SOS can prevent hydrolysis by pepsin by adhering to mucosal proteins, this surface enrichment of SOS might play an important role in the protection of the mucosa from refluxing acid or pepsin, almost as a preventive effect of SOS. It is known that sucralfate adheres specifically to inflammatory sites. In the present study, we confirmed that SOS also adheres to inflammatory lesions, similar to the actions of sucralfate under acidic conditions. Exposure of cationic mucosal proteins may cause SOS adhesion via an electrostatic interaction. These observations demonstrate efficient SOS delivery to the inflammatory oesophageal epithelial cells; thus, strengthening previously reported mechanisms^14^.

Our findings suggest that sucralfate may also be an effective option in patients without endoscopic manifestations, such as those with mild or grade A oesophagitis, or patients with non-erosive oesophageal reflux disease (NERD). In fact, two clinical trials have reported that sucralfate is effective for non-inflammatory diseases such as NERD and functional dyspepsia^29,30^. Visualisation of the formation of the thin SOS layer on the mucosa in this study provides supporting evidence for the potential application of sucralfate in non-inflammatory diseases.

In conclusion, the present study demonstrated that SOS administration protects against reflux oesophagitis via forming a thin SOS layer on the oesophageal mucosal surface. Since this layer formation is enhanced at inflammatory lesions, SOS and its polymer, sucralfate, can effectively protect the oesophageal mucosa against reflux oesophagitis.
